# THE EFFECT OF RETIREMENT TIMING ON HEALTH AND WELL-BEING OF HISPANICS COMPARED TO NON-HISPANIC WHITES LIVING IN THE U.S.

**DOI:** 10.1093/geroni/igz038.474

**Published:** 2019-11-08

**Authors:** Antonia E Diaz-Valdes Iriarte

**Affiliations:** Boston College School of Social Work, MA, United States

## Abstract

In the context of an aging society, where the proportion of older adults is rapidly increasing, ensuring healthier longer lives is key for individuals, families, policy makers and the population as a whole. In this context the productive aging framework has gained increased importance. There is evidence showing that engagement is related to late-life well-being and health (i.e., Hinterlong, 2006; Everard et al, 2000; Rozario et al, 2004; Matz-Costa et al, 2012). However, the productive aging framework lacks cultural sensitivity and evidence about the association between the effect of retirement on health and well-being in late-life is mixed. The current study seeks to contribute to this gap by exploring the consequences of the discrepancies between planned and actual retirement age on subjective health and well-being, comparing Hispanics and non-Hispanic Whites. A series of regression models were conducted to explore the effect of the discrepancy between planned and actual retirement age on retirement satisfaction, self-rated health and mental health (CESD). Results indicates that native born Hispanics presented more differences when compared to foreign born Hispanic than non-Hispanic Whites, which could indicate the effect of acculturation and its fading effect on cultural attitudes, such as familismo. Hispanic tend to have higher retirement satisfaction than non-Hispanics which is aligned with the happiness paradox found by Calvo and collagues (2017). Additionally, SES has a significant effect on health for non-Hispanic Whites but not among Hispanics. Finally, retirement timing predicted mental health among foreign born Hispanic but among native born Hispanics and non-Hispanic Whites.

